# GADD45B mediates podocyte injury in zebrafish by activating the ROS-GADD45B-p38 pathway

**DOI:** 10.1038/cddis.2015.300

**Published:** 2016-01-21

**Authors:** Z Chen, X Wan, Q Hou, S Shi, L Wang, P Chen, X Zhu, C Zeng, W Qin, W Zhou, Z Liu

**Affiliations:** 1 National Clinical Research Center of Kidney Diseases, Jinling Hospital, Nanjing University School of Medicine, Nanjing, China; 2 Department of Pediatrics and Communicable Diseases, University of Michigan Medical School, Ann Arbor, MI, USA

## Abstract

GADD45 gene has been implicated in cell cycle arrest, cell survival or apoptosis in a cell type specific and context-dependent manner. Members of GADD45 gene family have been found differentially expressed in several podocyte injury models, but their roles in podocytes are unclear. Using an *in vivo* zebrafish model of inducible podocyte injury that we have previously established, we found that zebrafish orthologs of *gadd45b* were induced upon the induction of podocyte injury. Podocyte-specific overexpression of zebrafish *gadd45b* exacerbated edema, proteinuria and foot-process effacement, whereas knockdown of *gadd45b* by morpholino-oligos in zebrafish larvae ameliorated podocyte injury. We then explored the role of GADD45B induction in podocyte injury using *in vitro* podocyte culture. We confirmed that GADD45B was significantly upregulated during the early phase of podocyte injury in cultured human podocytes and that podocyte apoptosis induced by TGF-*β* and puromycin aminonucleoside (PAN) was aggravated by GADD45B overexpression but ameliorated by shRNA-mediated GADD45B knockdown. We also showed that ROS inhibitor NAC suppressed PAN-induced GADD45B expression and subsequent activation of p38 MAPK pathway in podocytes and that inhibition of GADD45B diminished PAN-induced p38 MAPK activation. Taken together, our findings demonstrated that GADD45B has an important role in podocyte injury and may be a therapeutic target for the management of podocyte injury in glomerular diseases.

Podocyte dysfunction, injury or loss is a common and decisive cause of various glomerular diseases and understanding the molecular mechanism underlying podocyte response to stress will be very helpful to undermine the pathogenesis of podocyte injury and the targeted therapy for glomerular diseases.

The members of Gadd45 gene family, Gadd45a, Gadd45b and Gadd45r have been commonly implicated in stress signaling in response to physiological or environmental stressors, resulting in cell cycle arrest, DNA damage repair, cell survival, senescence and apoptosis.^1^ Recently, this gene family has been found differentially expressed in several podocyte injury models. Zhang *et al.*^2^ observed an induction of *GADD45β* mRNA expression by lipopolysaccharide in the lung, kidney and spleen, which had the highest *GADD45*β mRNA expression among all of the tissues examined. Jeffrey W Pippin reported that protein expression of GADD45 was increased in glomeruli from passive Heymann nephritis rats and cultured podocytes exposed *in vitro* to C5b-9. ^3^ More recently, Shi *et al.*^4^ reported that *Gadd45b* was upregulated in glomeruli of mice with podocyte-specific deletion of Dicer, suggesting the involvement of *Gadd45b* in podocyte injury. However, no functional characterization of *Gadd45* genes in podocytes has been conducted to date and the role of GADD45B in the context of podocyte injury remains unclear.

Zebrafish has emerged as a new vertebrate model system for renal glomerular research. The podocytes and renal glomeruli in zebrafish kidney are structurally, molecularly and functionally conserved, rendering zebrafish a valuable and relevant model for podocyte studies. To characterize the role of GADD45b in podocyte injury, we therefore employed zebrafish as an *in vivo* model system and human podocytes as an *in vitro* model. We observed the upregulation of GADD45B on podocyte injury in zebrafish renal glomeruli as well as in cultured human podocytes treated with TGF-β and PAN. We further showed that podocyte-specific overexpression of zebrafish orthologs of *gadd45b* predisposed podocytes to injury, whereas inhibition of *gadd45b* expression in zebrafish larvae ameliorated podocyte injury and reduced proteinuria. Furthermore, we found that the ROS-GADD45B-p38 pathway was involved in the regulation of GADD45B expression and deleterious role in podocyte injury. Collectively, we have identified GADD45B as an important player in podocyte injury.

## Results

### *gadd45ba/b* expression is upregulated in zebrafish with podocyte injury

**Figure 1:**
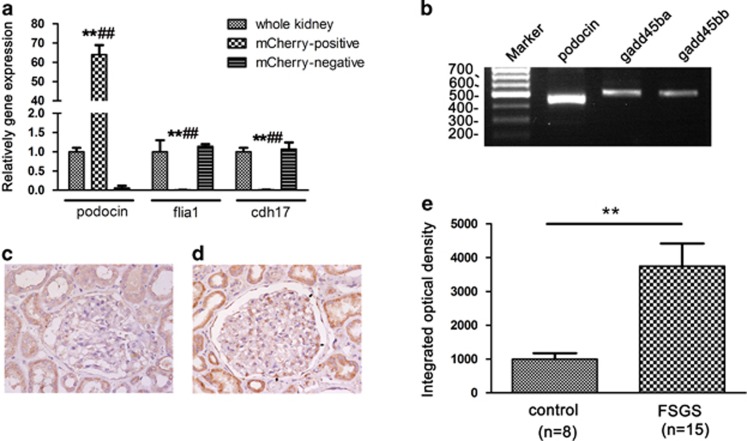
Validation of *gadd45ba/bb* expression on podocyte. (**a**) qRT-PCR validation of isolated podocytes. qRT-PCR analysis confirmed that the expression of podocyte marker podocin in isolated podocytes (mCherry positive) was highly enriched as compared with those from whole kidney and mcherry-negative cells. In contrast, expression of endothelial marker *flia1* and tubular marker *cdh17* were significantly lower in isolated podocytes as compared with those in whole kidney and mCherry-negative cells. Results are represented as mean±S.D. (*n*=3), **P<0.01 compared with whole kidney, ^##^*P*<0.01 compared with mcherry-negative cells. (**b**) RT-PCR analysis of *gadd45ba/bb* mRNA expression in isolated podocytes. *gadd45ba/bb* expressed in isolated podocytes which express *podocin* (**c**, **d**) GADD45B expression in biopsy of normal control (**c**) and FSGS patient (**d**) by immunohistochemical staining. GADD45B signals are very weak in glomeruli of normal kidney tissues, whereas GADD45B expression in FSGS patients was significantly increased as compared with normal control group. Arrows indicate GADD45B expression on podocytes of FSGS patient. Original magnification × 400 (**e**) Semiquantitative analysis of immunohistochemical staining results showed GADD45B expression of glomeruli of FSGS patients were significantly higher than the control group. Results are represented as mean±S.D. **P<0.01

We isolated podocytes from kidney of *Tg(pod:Gal4;UAS:NTR-mcherry)* zebrafish and confirmed the isolated podocytes had enriched podocin expression (Figure 1a). *gadd45ba/bb* mRNA expression were detected on the isolated podocytes (Figure 1b). We observed GADD45B expression on glomeruli of human biopsy and found that GADD45B signals are very weak in glomeruli of normal kidney tissues, whereas GADD45B expression in FSGS patients was significantly increased as compared with normal control group (Figures 1c and d).

**Figure 2:**
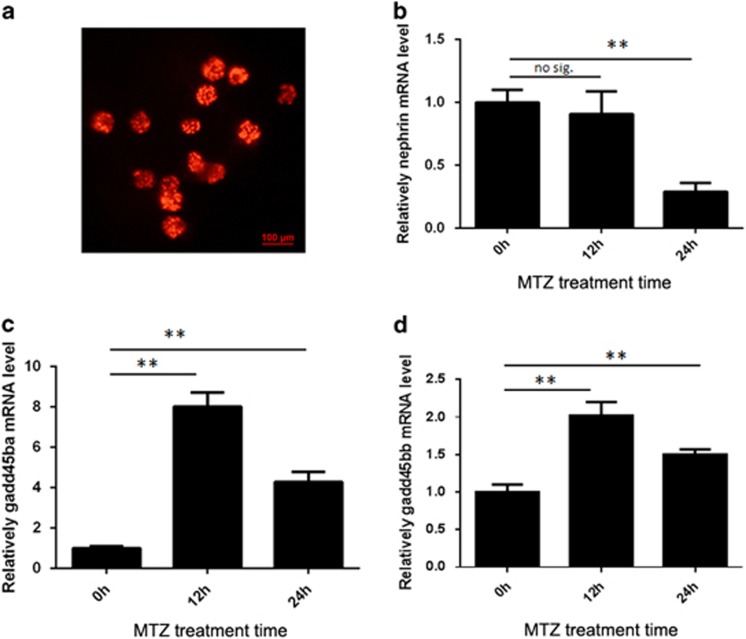
*gadd45ba* and *gadd45bb* are induced by podocyte Injury in zebrafish. (**a**) Dissected renal glomeruli from *Tg(pod:gal4;UAS:NTR-mcherry*), mCherry expression in podocytes allow for easy collection under fluorescence microscope. (**b–d**) Quantitation of nephrin (**b**), gadd45ba (**c**) and gadd45bb (**d**) transcripts by quantitative RT-PCR before and after MTZ (120 *μ*M) treatment. Results are represented as mean±S.D. (*n*=3), **P<0.01

Previously, we have established a transgenic zebrafish model of inducible podocyte injury, in which a bacterial nitroreductase (NTR) is specifically expressed under the control of zebrafish podocin promoter.^5^ In this transgenic zebrafish, metronidazole (MTZ) treatment can induce podocyte foot-process effacement, podocyte apoptosis, edema and proteinuria. Here, we refined this model of podocyte injury using a double transgenic zebrafish *Tg(pod:Gal4;UAS:NTR-mCherry)*, in which transcription factor *Gal4* was specifically expressed in podocytes, and NTR-mCherry was under the control of *UAS* promoter. In this model, renal glomeruli could be conveniently isolated based on the mCherry fluorescence and podocyte injury induced by MTZ treatment (Figure 2a). We treated the double transgenic zebrafish with MTZ for 12 and 24 h followed by the isolation of zebrafish glomeruli under fluorescence microscopy. qRT-PCR results showed that the mRNA expression of both zebrafish orthologs of *GADD45B* (*gadd45ba and gadd45bb*) were significantly upregulated at as early as 12 h post treatment, when nephrin expression was barely decreased (Figures 2b–d). After 24 h of MTZ treatment, nephrin expression was significantly decreased, whereas the elevated mRNA expression of both *gadd45ba* and *gadd45bb* persisted. Thus, *gadd45b* was induced at the early phase of podocyte injury prior to the downregulation of *nephrin*, suggesting it is an early mediator of podocyte injury. In addition, we found that *gadd45ba* expression was more abundant than *gadd45bb* in normal zebrafish glomeruli and the induction of *gadd45ba* by podocyte injury was more prominent than that of *gadd45bb* (Figures 2c and d), suggesting *gadd45ba* may have a major role in zebrafish podocytes compared with *gadd45bb*.

### *gadd45ba/b* overexpression aggravates MTZ-induced podocyte injury and proteinuria

**Figure 3:**
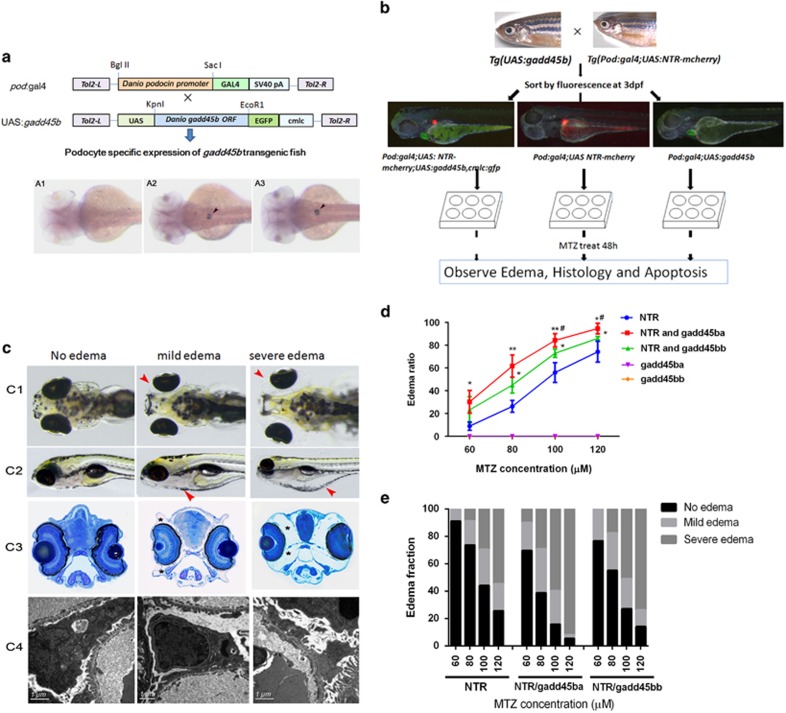
*gadd45ba/b* overexpression in podocytes aggravates MTZ-induced podocyte injury. (**a**) Schematic graph showing the generation of transgenic fish. Podocyte-specific expression of *gadd45ba/b* is confirmed by *in situ* hybridization in WT (A1), *Tg* (pod:Gal4,UAS:gadd45ba) (A2) and *Tg* (pod:Gal4,UAS:gadd45bb) (A3) at 3dpf. (**b**) Experimental scheme to test *gadd45ba/b* overexpression on MTZ-induced podocyte injury. (**c**) Representative figures showing the phenotypes due to MTZ-induced podocyte injury. (**c**1) Dorsal view of five dpf larvae showing periorbital edema (red arrowhead). (**c**2) side view of five dpf larvae showing whole-body edema (red arrowhead). (**c**3) Cross-section of eyes of five dpf larvae stained with methylene blue showing the severity of periorbital edema (asterisks). (**c**4) TEM showing the degree of foot-process effacement. (**d**) Quantitation of the percentage of larvae with periorbital edema. The nonfluorescence fish were used as negative controls and had no observable periobital edema phenotypes after being treated with MTZ. (**e**) Quantitation of the percentage of larvae with mild and severe edema. Results are represented as mean±S.D. (*n*=3), *P<0.05, **P<0.01 *versus* NTR group, ^#^*P*<0.05 *versus* NTR and gadd45bb group

Given that *gadd45b*a*/b* expression was induced in zebrafish podocyte injury, we sought to functionally characterize *gadd45b*a*/b* in podocyte injury. First, we generated transgenic zebrafish lines stably expressing *gadd45ba* or *gadd45bb* in podocytes using the binary Gal4/UAS system (Figure 3a). GFP expression in cardiomyocytes allowed for easy identification of the *Tg(UAS:gadd45ba/b,cmlc2:GFP)* transgenic fish. When *Tg(UAS:gadd45ba/b,cmlc2:GFP)* was mated with *Tg(pod:Gal4;UAS:NTR-mCherry)*, podocin-promoter-driven Gal4 expression could activate both *NTR-mCherry* and *gadd45ba/b* expression exclusively in podocytes, which was confirmed by whole-mount *in situ* hybridization (Figure 3a).

The transgenic fish *Tg(pod:Gal4;UAS:NTR-mCherry;UAS:gadd45ba/b,cmlc2:GFP)* appeared normal and fertile, without any edema. To determine the role of *gadd45ba/b* in podocyte injury, we then induced podocyte injury with MTZ after sorting the transgenic embryos by the expression of fluorescent proteins. Larvae with GFP fluorescence in the heart and mCherry fluorescence in glomeruli expressed both *gadd45ba/b* and NTR-mCherry in podocytes, whereas larvae with mCherry fluorescence in glomeruli only expressed NTR-mCherry in podocytes and hence as controls (Figure 3b). As MTZ-induced podocyte injury causes a very unique periorbital edema (POE) phenotype in zebrafish larvae, resembling nephrotic syndrome in human patients (Figure 3c, Supplementary Figure 1), we categorized the fish with POE into two subgroups according to the severity of edema. The mild group had mild POE alone with moderate foot-process effacement but the severe group displayed severe POE and whole-body edema with more extensive foot-process effacement (Figure 3c). By quantifying the percentage of zebrafish larvae with POE, we found that MTZ could induce POE in zebrafish carrying NTR-mCherry in a dosage-dependent manner. In addition, *gadd45ba/b* overexpression in zebrafish podocytes significantly increased the penetrance of MTZ-induced POE by ~20% (Figure 3d,Supplementary Table 1). Indeed, *gadd45ba/b* overexpression in podocytes significantly aggravated the severity of edema induced by MTZ (Figure 3e). Although the percentage of mild edema group was not significantly changed, the percentage of severe edema group was drastically increased by *gadd45ba/b* overexpression. It is also notable that the effect of *gadd45ba* overexpression was more prominent than that of *gadd45bb* overexpression (Figures 3d and e). In conclusion, excessive *gadd45ba/b* in podocytes aggravated podocyte injuries.

**Figure 4:**
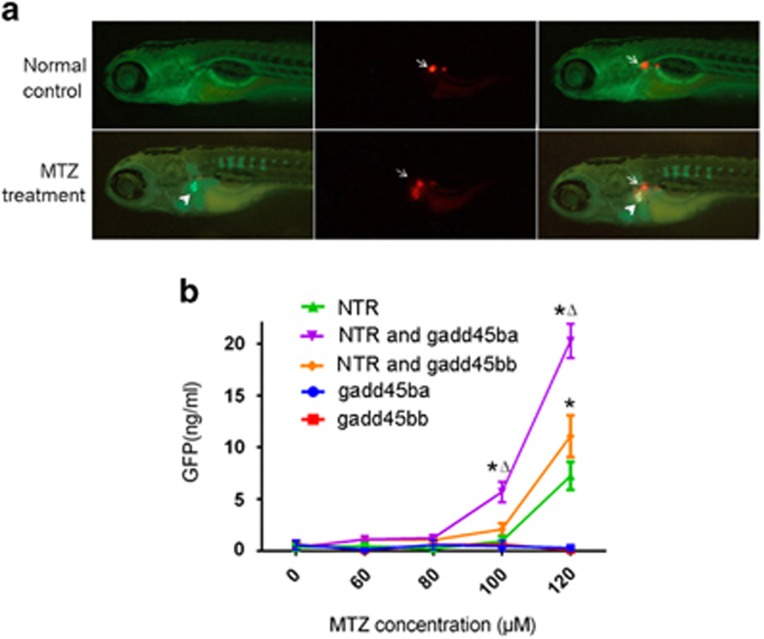
*gadd45ba* and *gadd45bb* overexpression in podocytes aggravates MTZ-induced proteinuria. (**a**) Representative fluorescence figures showing disruption of the glomerular filtration barrier by MTZ leads to accumulation of GFP fluorescence in the proximal tubules (arrowheads) in *Tg(pod:Gal4;UAS-NTR-mCherry; lfabp:VDBP-GFP)*. mCherry fluorescence (arrows) is reduced in glomeruli and accumulated in proximal tubules presumably due to podocytes loss. (**b**) Quantitation of GFP by ELISA showing zebrafish expressing *gadd45ba/b* in podocytes exhibit more poteinuria induced following MTZ-induced podocyte injury. Results are represented as mean±S.D. (n=3), *P<0.01 *versus* NTR group; Δ *P*<0.01 *versus* NTR and gadd45bb group

To test whether overexpression of *gadd45ba/b* exacerbates proteinuria, we performed proteinuria assay using a previously established *Tg(lfabp:VDBP-GFP)* zebrafish expressing GFP-tagged vitamin D-binding protein (VDBP).^5^ In this transgenic fish, VDBP-GFP, which has a molecular weight (MW) and isoelectric point (PI) (MW:79.6 kD; PI 5.67) comparable to human albumin (MW:65.5; PI: 5.97), serves as a tracer plasma protein for both size and charge selection of glomerular filtration barrier. Disruption of the glomerular filtration barrier by MTZ-induced podocyte injury in *Tg(pod:Gal4; UAS-NTR-mCherry; lfabp:VDBP-GFP)* led to strong accumulation of GFP fluorescence in the proximal tubules, as VDBP-GFP is reabsorbed by tubular epithelium (Figure 4a). Meanwhile, mCherry fluorescence was present in the proximal tubules, presumably due to the tubular reabsorption of mCherry protein released from disintegrated podocytes (Figure 4a). Using ELISA against GFP to measure the excretion of VDBP-GFP into water by the transgenic larvae, we found that overexpression of *gadd45ba/b* in podocytes significantly increased proteinuria induced by 24 h of MTZ treatment (Figure 4b, Supplementary Table 2). Noticeably, without MTZ treatment, *Tg(pod:Gal4;UAS:NTR-mCherry;UAS:gadd45ba/b;lfabp:VDBP-GFP)* did not show any significant excretion of GFP either, indicating that *gadd45ba/b* overexpression alone was not sufficient to impair the glomerular filtration barrier. These proteinuria data were consistent with the tubular accumulation of GFP and the edema phenotype.

### *gadd45ba/b* overexpression aggravates MTZ-induced podocyte apoptosis

**Figure 5:**
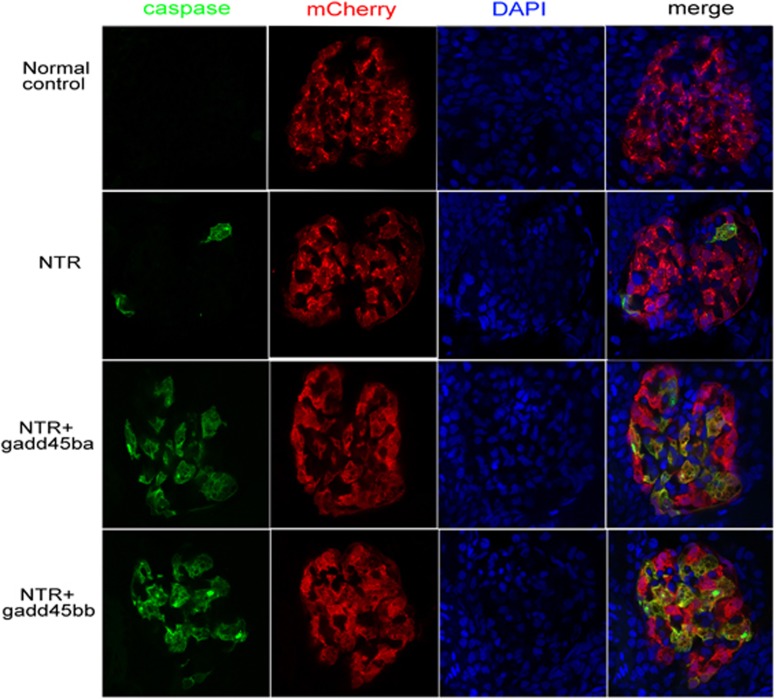
*gadd45ba/gadd45bb* overexpression aggravated podocyte apoptosis. Confocal images of the pronephric glomeruli in zebrafish embryos were treated with 100 *μ*M MTZ from 72 hpf to 96 hpf. Green fluorescence represents active caspase-3 staining; podocytes are marked with mCherry red fluorescence and nuclei are labeled with DAPI (original magnification, × 630)

To investigate the effect of *gadd45ba/b* overexpression on podocyte apoptosis, we performed immunostaining of cleaved caspase-3, an essential mediator of apoptosis, on zebrafish pronephric glomeruli. We observed that apoptosis was significantly increased following MTZ treatment in the pronephric glomeruli of *gadd45ba/b* overexpression fish than those of the control group expressing NTR-mCherry alone in podocytes (Figure 5).

### Inhibition of *gadd45ba/b* expression alleviates MTZ-induced podocyte injury and proteinuria

**Figure 6:**
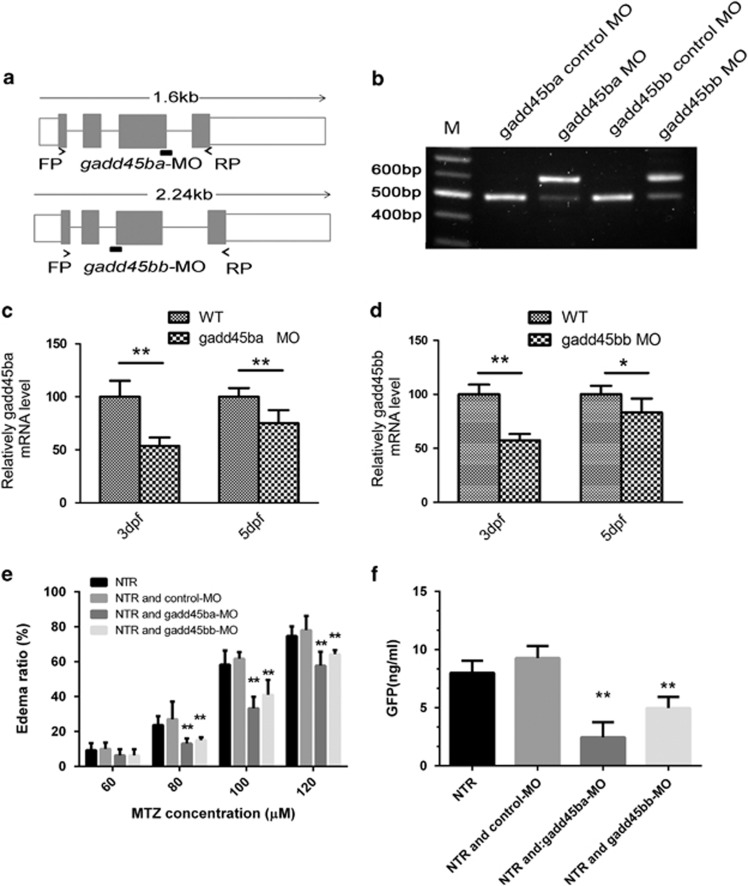
Inhibition of *gadd45ba* or *gadd45bb* expression alleviates MTZ-induce edema and proteinuria. (**a**) Genomic organization of zebrafish gadd45ba and gadd45bb; target sites of the splice morpholinos and positions of primers used to determine the efficacy of MOs. (**b**) RT-PCR analysis of gadd45ba/bb mRNA in uninjected and gadd45ba/bb-splice-MO-injected embryos at 5 dpf. (**c**, **d**) qRT-PCR quantify of *gadd45ba/bb* mRNA level in wild-type fish and gadd45ba/bb-splice-MO-injected embryos at 3dpf and 5dpf. *P<0.05, **P<0.01. (**e**) Quantitation of the periorbital edema phenotype. (**f**) Quantitation of proteinuria with ELISA for GFP. *P<0.05, **P<0.01 *versus* NTR group

Based on the aforementioned results, we wondered whether inhibition of *gadd45ba/b* in zebrafish could reduce MTZ-induced podocyte injury. To answer this question, we utilized morpholino-oligos specifically blocking the mRNA splicing of either *gadd45ba* or *gadd45bb* (Figure 6a). The efficacy of these MOs was confirmed by RT-PCR and sequencing, showing that gadd45ba-MO and gadd45bb-MO could block the splicing of adjacent introns (Figure 6b). qRT-PCR results showed that gadd45ba-MO resulted in 47.1±6.8% (3dpf) and 25.3±8.5% (5dpf) reduction in *gadd45ba* mRNA level, whereas gadd45bb-MO resulted in 42.4±5.9% (3dpf) and 19.2±7.6% (5dpf) in *gadd45bb* mRNA level (Figures 6c and d). Injection of either gadd45ba-MO or gadd45bb-MO did not cause any embryonic developmental defect prior to the induction of podocyte injury. gadd45ba-MO or gadd45bb-MO could, however, significantly alleviate MTZ-induced POE while control-MO did not impose such an effect (Figure 6e,Supplementary Table 3). Interestingly, the effect on podocyte injury by *gadd45ba* knockdown is more pronounced than *gadd45bb*, consistent with the finding that overexpression of *gadd45ba* had a greater effect in aggravating podocyte injury than *gadd45bb*. Furthermore, knockdown of *gadd45ba/b* could also significantly lower the MTZ-induced proteinuria in our zebrafish models (Figure 6f, Supplementary Table 4).

### GADD45B overexpression sensitizes podocytes to TGF-β and PAN-induced apoptosis

**Figure 7:**
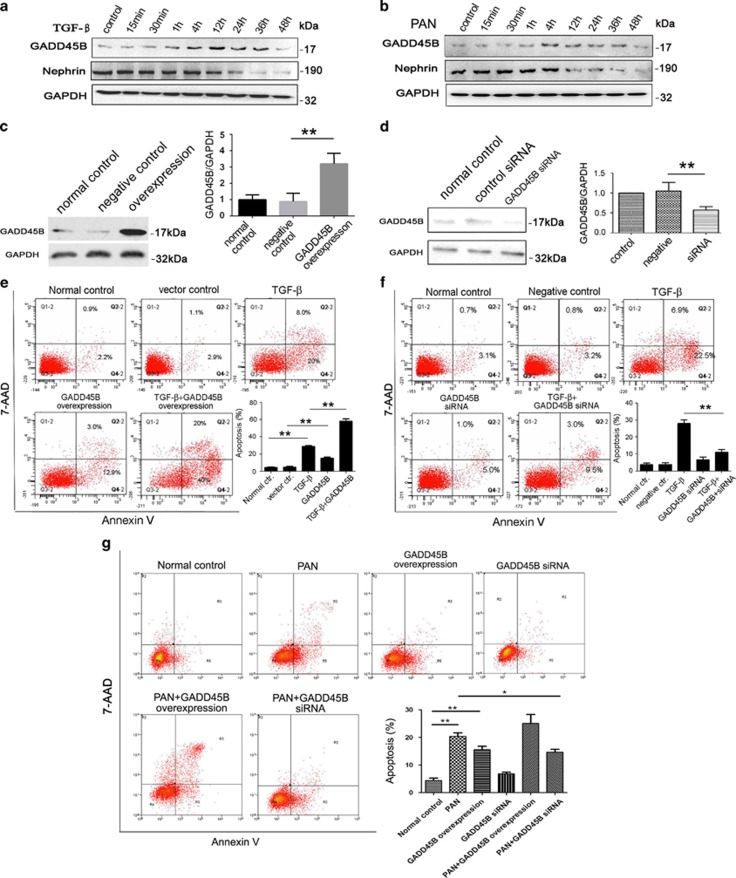
GADD45B expression are upregulated by TGF-*β* and PAN in cultured human podocytes and podocyte apoptosis induced by TGF-*β* is aggravated by GADD45B overexpression and ameliorated by GADD45B knockdown. (**a**, **b**) Western blot analyses of GADD45B and nephrin protein expression upon (**a**) TGF-*β* (5 ng/ml) and (**b**) PAN (50 *μ*g/ml) stimulation. Relative protein abundance of each blots was normalized to the gray value of GAPDH. (**c**, **d**) Western blot showing GADD45B overexpression (**c**) or knocked down by shRNA (**d**) in cultured human podocytes. (**e**–**g**) Annexin V staining followed by FCM showing podocyte apoptosis induced by TGF-*β* (**e**, **f**) and PAN (**g**) is aggravate by GADD45B overexpression and ameliorated by GADD45B knockdown. Relative protein abundance of each blot was normalized to the gray value of GAPDH. Results are represented as mean±S.D. (*n*=3). *P<0.05, **P<0.01

Podocyte deleterious factors, such as TGF-*β* and PAN can induce GADD45B expression, which preceded the decrease of NEPHRIN protein level induced by TGF-*β* and PAN treatment (Figures 7a and b). To determine the effect of GADD45B upregulation on podocyte injury, GADD45B was overexpressed in cultured human podocytes, which was confirmed by western blot (Figure 7c). Podocytes with GADD45B overexpression had a significantly higher percentage of Annexin V-positive apoptotic cells compared with the mock-transfected control cells in the absence or presence of TGF-β or PAN, suggesting that GADD45B contributes to apoptosis in podocytes (Figures 7e and g). Consistently, when GADD45B expression was knocked down using the GADD45B shRNA, confirmed by western blot (Figure 7d), TGF-β or PAN-induced podocyte apoptosis was significantly decreased compared with shRNA control group or normal control group (Figures 7f and g).

### GADD45B upregulation is associated with ROS generation and p38 MAPK pathway activation in podocytes

**Figure 8:**
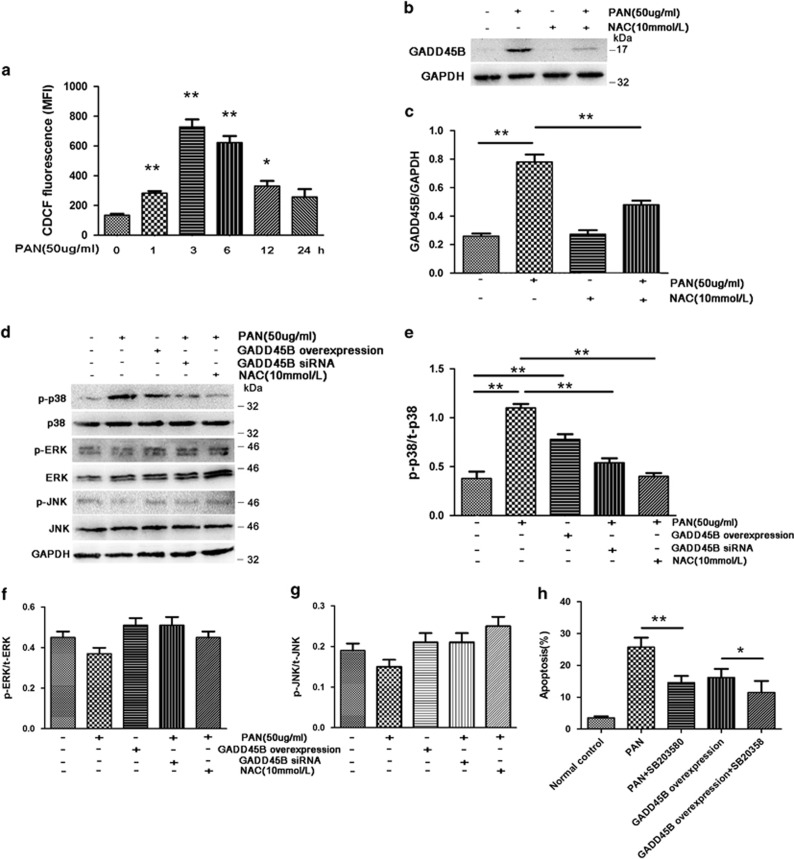
ROS induced by PAN upregulates GADD45B, which subsequently activates p38 MAPK pathway in cultured human podocytes. (**a**) Time course of PAN-induced cellular ROS generation. (**b**) Western blot analyses of PAN-induced GADD45B upregulation was diminished by ROS inhibitor NAC. (**c**) Quantitation of GADD45B protein level in podocytes treated with PAN and NAC. (**d**) PAN treatment and GADD45B overexpression activates p38 MAPK pathway in cultured podocytes and knockdown of GADD45B inhibits the activation of p38 MAPK pathway by PAN. In contrast, the above treatments have no significant effect on phospho-ERK and phospho-JNK MAPK levels. (**e**–**g**) Quantitation of p38(**e**), ERK(**f**) and JNK(**g**) phosphorylation in cultured podocytes (**h**) p38 inhibitor SB-203580 (25 *μ*mol/l) inhibited PAN and GADD45B overexpresson induced podocyte apoptosis. *P<0.05, **P<0.01. Relative protein abundance of each blot was normalized to the gray value of GAPDH. Results are represented as mean±S.D. (*n*=3). *P<0.05, **P<0.01

Given that GADD45B was upregulated by multiple factors related to oxidative stress in podocytes and that the cytotoxicity of MTZ/NTR system is related to the reduction of the nitro group to nitro radical anion,^6^ we deduced that reactive radicals may be involved in the upregulation of GADD45B in injured podocytes. As overproduction of ROS has been shown to cause podocyte injury induced by PAN toxicity,^7, 8, 9^ we tested whether oxidative stress-mediated GADD45B upregulation in PAN-induced podocyte injury. PAN (50 *μ*g/ml) significantly increased ROS generation in podocytes during 30 min to 12 h post treatment (Figure 8a), whereas the same PAN treatment induced GADD45B expression by nearly threefold at 12 h post treatment (Figures 8b and c). Pretreatment of podocytes with antioxidant N-acetylcysteine (NAC, 10 mmol/l) before PAN exposure led to a significant reduction in GADD45B level (Figures 8b and c), suggesting the production of ROS mediated the upregulation of GADD45B in podocytes.

GADD45B interacts with MAPK upstream kinase MTK1/MEKK4, leading to the activation of p38 or c-jun N-terminal kinase signaling pathways.^10, 11^ MAPKs are known to have crucial roles in the progression of various glomerulopathies, and their inhibition is emerging as a promising therapeutic area for renal diseases.^12^ We found that GADD45B overexpression induced phosphorylation of p38 MAPK in podocytes (0.75±0.09 *versus* 0.37±0.10 normal control, *P*=0.008) (Figures 8d and e), but had no effect on activation of JNK and ERK phosphorylation (Figures 8d, f and g). Knockdown of GADD45B expression by shRNA diminished PAN-induced p38 MAPK phosphorylation (0.55±0.08 *versus* 1.13±0.08 PAN, *P*=0.0009). In addition, p38 inhibitor SB-203580 (25 *μ*mol/l) effectively inhibited PAN and GADD45B overexpression induced podocyte apoptosis by 43.1±9.7% and 29.1±5.3%, respectively (Figure 8h). Thus, our data suggest that the ROS-GADD45B-p38 axis mediates the podocyte injury.

## Discussion

GADD45B is among a group of genes upregulated in response to stressful growth arrest or DNA damage.^1, 13^ They are involved in many processes during cellular adaptation to a diverse array of cellular stresses, including apoptosis, DNA repair, chromatin regulation and cell cycle delay.^1, 14, 15, 16, 17^ However, the role of GADD45B in cell stress response is rather complex: it may exert protective or deleterious effects depending on the type of cells and insults. For example, Gupta *et al.*^18^ found that *Gadd45b* protected hematopoietic cells from DNA-damaging agents, including ultra violet-induced apoptosis. Larsen *et al.*^19^ reported that IL-1*β* stimulated the time-dependent induction of endogenous Gadd45b in rat insulinoma INS-1E cells and rat islets and Gadd45b overexpression can significantly reduce apoptosis induced by IL-1*β* in INS-1E cells and in mouse beta-TC3 cells. On the other hand, Kim *et al.*^20^ demonstrated that Gadd45*β-*mediated cardiomyocyte apoptosis under ischemia/anoxia both in cultured H9c2 cells and in the rat heart *in vivo*. Given the recent studies of a number of animal models of podocyte injury and glomerular diseases implicating GADD45B in podocyte injury, we now have utilized zebrafish models and cultured human podocytes to determine that the induction of GADD45B is an early response to podocyte injury that regulates podocyte apoptosis in response to ROS through the activation of p38 MAPK pathway.

The podocyte function in zebrafish has been proven to be highly conserved in terms of its essential role in maintaining the glomerular filtration barrier. Previous studies of zebrafish orthologs of human nephrosis genes have also demonstrated that these genes have a highly conserved podocyte-specific function across species.^21, 22^ Therefore, it is reasonable to speculate the molecular mechanisms underlying podocyte injury responses are also conserved in zebrafish. In this study, we utilized the zebrafish models that we have previously established to facilitate our functional study of GADD45B in podocytes *in vivo*.^5, 6^ In fact, we observed an induction of *gadd45ba/b* in zebrafish pronephric glomeruli following induced podocyte injury. This is consistent with previously published data from rodent models of podocyte injury^3, 4^ and was confirmed in cultured human podocytes injured with various deleterious factors by us. Taking advantage of its convenience and versatility in genetic manipulation, we generated new lines of transgenic zebrafish to demonstrate that overexpression of *gadd45ba/b* in podocytes exacerbates podocyte apoptosis and proteinuria, whereas inhibition of gadd45ba/b reduces podocyte injury and proteinuria. We designed splice-blocking morpholinos to avoid early embryonic developmental defects due to blocking the function of maternally deposited gadd45b.^10^ This allowed us to test the specific function of injury-induced gadd45b in podocytes.

Taken together, our zebrafish data not only produce *in vivo* characterization of *gadd45b* function in podocytes but also suggest that zebrafish pronephros can be used as an experimental platform for effective functional studies of podocyte-specific genes. Particularly, the toxicity of MTZ as an antibiotic prodrug is mediated by the reduction of the nitro group into protonated one electron nitro radical anion to oxidized DNA,^6^ so our MTZ-induced podocyte injury model is comparable to the conventional podocyte injury models for rodents, such as PAN and adriamycin, which also impose oxidative stress and DNA damage to podocytes.^7, 8, 9^ TGF-*β* was also found to affect tubular epithelia cell and kidney fibroblast through oxidative stress.^23, 24^

We further explored the mechanism underlying GADD45B induction and subsequent podocyte injury using the *in vitro* human podocyte culture. Pretreatment of podocytes with antioxidant NAC significantly blocked the induction of GADD45B by PAN, supporting that oxidative stress is correlated with the upregulation of GADD45B in injured podocytes. Moreover, GADD45B overexpression activated p38 MAPK pathway and GADD45B downregulation diminished PAN-induced p38 MAPK activation. P38 inhibitor SB-203580 reduced GADD45B overexpression induced podocyte damage. Among the many kinases activated by ROS, mitogen-activated protein kinases, p38 and c-Jun NH2-terminal kinase have been shown to have a key role in oxidative cell injury^25, 26^ and p38 activation is crucially involved in podocyte injury, regulating podocyte skeleton disruption, autophagy, apoptosis and so on.^27, 28, 29^ Our data established a connection from ROS to p38 activation through GADD45B, elucidating one of the molecular pathways that contribute to podocyte injury.

We identified GADD45B as a new player involved in podocyte stress response in zebrafish as well as in human podocytes. We have also presented successful example of functional analyses of genes involved in podocyte injury using zebrafish models. In particular, with the new genetic tools (CRISPR/Cas9 and TALEN and so on) and transgenic models available for zebrafish podocyte research (inducible podocyte injury and proteinuria assay and so on), the usefulness of this model organism should not be confined to developmental biology of kidney, but rather can be expanded into the study of genetic regulation of podocyte injury and disease.

## Materials and Methods

### Fish breeding and maintenance

Zebrafish (*Danio rerio*) were reared and maintained following standard procedures.^30^ Embryos were collected after natural spawn, kept at 28.5 °C, and staged as hours (hpf) or days (dpf) post fertilization. All procedures were approved by University Committee on Use and Care of Animals at the University of Michigan.

### DNA Constructs and transgenic lines

Transgenic fish lines *Tg(pod:Gal4)*, *Tg(UAS:gadd45ba,cmlc2:gfp)* and *Tg(UAS:gadd45bb,cmlc2:gfp)* were generated using Tol2 transposon constructs and the Tol2 gateway kit.^31, 32^ For pod: Gal4, EGFP was replaced by coding sequence of Gal4 in the pod:EGFP plasmid described previously at the AgeI-ClaI restriction sites. *Danio gadd45ba* and *gadd45bb* cDNA was amplified by RT-PCR from 24 hpf zebrafish embryos. The amplicon were cloned into pME-MCS and sequenced for verification. The primers for *gadd45b*a are sense: 5′-ttGGTACCatgaccctggaagaagtcgttg-3′, antisense: 5′-aGAATTCtcagcgttcttgcagggacag-3′. The primers for *gadd45bb* are sense: 5′-ttGGTACCatgactctggaggaagttgttg-3′, antisense: 5′-aGAATTCtcagcgctcttgcagg-3′. The middle-entry clones, p5E-UAS and p3E-cmlc2:EGFP-pA were assembled using the LR Clonase II plus according to the manufacturer's protocol (Invitrogen, Grand Island, NY). *Tg (UAS:NTR-mcherry)* was obtained from Zebrafish International Resource Center (ZFIN ID: ZDB-TGCONSTRCT-110215-5).^33^

### Microinjection and transgenic fish screening

Expression constructs, together with the Tol2 transposase RNA, were co-injected into one-cell stage AB strain zebrafish embryos as previously described.^34^ The embryos were maintained in egg water in incubator at 28.5 °C. All of the injected embryos were raised to sexual maturity and screened for germline transmission. For detection of *Tg(pod:Gal4)* transgenic founder fish, genomic DNA from pools of F_1_ embryos (24 hpf) was isolated accordingly.^30^ PCR was carried out using gene-specific genotyping primers for pod:Gal4: sense:5′- CTGGGAGTGTCGCTACTCTC-3′, antisense 5′- CTACATATCCAGAGCGCCGT-3′ for UAS:NTR-mcherry, sense 5′- ATATACCGGTCAAGCTTAGGCCTCCAAGGC-3′, antisense 5′- ATATATCGAT TGGATCCCAAACAGACGCGTC-3′. To identify *Tg(UAS:gadd45ba:cmlc2:GFP)* and *Tg(UAS:gadd45bb:cmlc2:GFP)* transgenic founders, transgenic fish was crossed with wild-type fish (AB*), F_1_ embryos (72 hpf) were screened under fluorescence microscopy. Embryos with green fluorescence in heart were raised to adulthood.

Tg(*pod:Gal4*) F1 transgenic fish crossed with *Tg(UAS:NTR-mCherry)* and raised embryos with mCherry expression in pronephros to adulthood to obtain double transgenic fish *Tg(pod:Gal4;UAS:NTR-mcherry).*

### Podocyte isolation

Kidney from *Tg(pod:Gal4;UAS:NTR-mcherry)* adult zebrafish were manually dissected and dissociated in 0.5% trypsin/collagenase. Dissociated cells were then filtered through a 70-*μ*m strainer and filtered again through a 30-*μ*m strainer. For the purification of podocytes,^35^ kidney single cells were incubated with Anti-mCherry monoclonal antibody (EarthOx, Millbrae, CA, USA) (1 : 200) coated Dynabeads pan mouse IgG (Invitrogen dynal, Oslo, Norway) at 4 °C. The positive and negative fractions were collected as mCherry-positive and mCherry-negative fractions, respectively. Isolated cells were placed in RLT Buffer (Qiagen, Hilden, Germany) and RNA was extracted using the RNeasyMicro Kit (Qiagen).

### Induction of podocyte injury

*Tg(pod:Gal4; UAS:NTR-mcherry)* was crossed with *Tg(UAS:gadd45ba,cmlc2:GFP)* or *Tg(UAS:gadd45bb,cmlc2:GFP)*. In all, 80hpf embryos were screened with fluorescence microscopy and separated into three groups, (1) embryos with GFP fluorescence (green) in heart and mCherry fluorescence (red) in pronephros. These embryos overexpress both *gadd45b* and *NTR-mCherry* in podocytes; (2) embryos with mCherry fluorescence (red) in pronephros only. These embryos expresses NTR-mCherry in podocytes; (3) embryos without fluorescence, this group served as negative control.

Metronidazole (1-[2-hydroxyethyl]-2-methyl-5-nitroimidazole) is reduced by *E. coli* NTR and converted into a potent DNA interstrand cross-linking molecule, causing cell death. To induce podocyte-specific injury, MTZ was added to E3 medium together with 0.1% DMSO. Embryos sorted at 80hpf were treated with MTZ/DMSO at the designated dosages for 48 h. In total, 0.1% DMSO was used as the vehicle control in all experiments. The experiment design is showed in Figure 7a.

### Reverse transcription PCR

Zebrafish Glomeruli were dissected from *Tg(pod:Gal4;UAS-NTR-mCherry)* and total RNA was isolated with RNA miniprep (Zymoresearch, Orange County, CA, USA). First-strand cDNAs were synthesized using the Superscript II first-strand synthesis system (Invitrogen), following the manufacturer's protocol. In total, 2 μl of the reserve transcription product was used for PCR with gene-specific primers: for gadd45ba (sense) 5′-ACTGCATCCTCGTCACTAACTC-3′ and (antisense) 5′-TTTTGCAACGGCTCTCCTCA-3′ for gadd45bb (sense) 5′-TGTTACTAACCCCCAAGCCG-3′ and (antisense) 5′-GGCAATAGAAGGCACCCAC-3′. The qRT-PCR analysis to determine the expression levels of gadd45b genes was performed using the Power SYBR Green PCR Master Mix (Applied Biosystems, Rockford, IL, USA). The levels of *ef1a* were used to normalize the relative mRNA abundance.

### Whole-mount *in situ* hybridization

Whole-mount *in situ* hybridization using digoxigenin (DIG)-labeled riboprobes was carried out as previously described.^36^ The embryos were fixed with 4% paraformadehyde (PFA) in phosphate-buffered saline (PBS), hybridized with a DIG-labeled gadd45b RNA probe at 70 °C, followed by incubation with anti-DIG antibody conjugated with alkaline phosphatase and by staining with the substrates, nitro blue tetrazolium and 5-bromo, 4-chloro, 3-indolyl phosphate (NBT/BCIP).

### Apoptosis detection by caspase-3 immunostaining

Antibody against cleaved caspase-3 (BD, Franklin Lakes, NJ, USA) was used at 1 : 500 and secondary antibody was Alexa fluo488 labeled antibody (Invitrogen, Eurgen, OR, USA) at 1 : 2000. The confocal images were captured with a Leica SP5 laser scanning microscope.

### Proteinuria assay

Transgenic fish were subjected to the induction of podocyte injury aforementioned at specified developmental stages. To measure GFP leakage with ELISA, 20 embryos per well were placed into 12-well plates at 80hpf and treated with MTZ in E3 medium for 24 h and 100 ml of medium was assayed using the GFP ELISA kit (Cell Biolabs, San Diego, CA, USA) following the manufacturer's protocol. Each experiment was repeated for at least three times independently.

### Transmission electron microscopy

In all, 96hpf embryos were fixed in cold 3.75% glutaraldehyde for 4 h and postfixed in phosphate-buffered 1% osmium tetroxide for 2 h. Followed by dehydration with graded series of ethanol, the specimens were embedded in Epon 812 Ultrathin sections (70 nm), were stained with uranyl acetate for 20 min and subsequently stained with lead citrate for 15 min at room temperature. The specimens were examined and photographed with Hitachi 7500 transmission electron microscope (Tokyo, Japan).

### Morpholino antisense oligonucleotides

Morpholino antisense oligonucleotides (MOs) were designed against splice-blocking sequence of zebrafish *gadd45ba* and *gadd45bb*. The MOs were obtained from GENE TOOLS, LLC, Philomath, OR, USA. The following sequences of morpholinos were used: *gadd45ba* (Exon 3 Splice Blocking) 5′-TGTATGTGTCTACTTACAGTGACGA-′3. *gadd45bb* (Exon 3 Splice Blocking) 5′-ATCCCTGAAGAAGTTGACAAACGCA-′3 and Control oligo: 5′CCTCTTACCTCAGTTACAATTTATA-3′. The morpholinos were diluted with the injection solution containing 100 mM KCl and 10 mM HEPES (pH 7.6) and were injected at a final concentration of 0.5 mM (*gadd45ba-MO*) and 0.75 mM (*gadd45bb-MO*) into one-cell stage embryos. For each morpholino, >300 individual embryos were injected. Splice blocking was verified using RT-PCR. The primer sets used were: *gadd45ba* sense, 5′-ttGGTACCatgaccctggaagaagtcgttg-3′, antisense: 5′-aGAATTCtcagcgttcttgcagggacag-3′. *gadd45bb* sense: 5′ ttGGTACCatgactctggaggaagttgttg-3′, antisense: 5′- aGAATTCtcagcgctcttgcagg-3′. PCR products were gel-purified and sequence verified.

### Human podocyte culture, treatment and transfection

Human podocytes were cultured as described.^37^ In brief, cells were grown at the permissive temperature (33 °C) in RPMI 1640 medium supplemented with 10% fetal bovine serum, insulin-transferrin-selenium, glutamine and penicillin/streptomycin (all from Life Technologies, Carlsbad, CA, USA). To induce differentiation, cells were switched to the non-permissive temperature (37 °C) for 14 days. The podocytes were treated with 5 ng/ml TGF-*β*, 50 *μ*g/ml PAN to induce injury.

*GADD45B* stable podocytes transfectants were generated. Full-length *GADD45B* cDNA was cloned into the pLenti6.3-IRES2-eGFP vector (Invitrogen, Shanghai, China) to generate pLenti6.3-*GADD45B*-IRES2-eGFP. After confirmed by sequencing, the vector and its helper vectors were mixed and co-transfected into 293 T cells to obtain recombinant virus containing *GADD45B* gene, which were used to infect proliferating podocytes. Viruses only carrying eGFP were used as control. Stably transfected podocytes were selected in serial passages using blasticidin (10 *μ*g/ml). Stably transfected podocytes were allowed to differentiate as described above, except in the presence of lower concentrations of blasticidin (2 *μ*g/ml).

### Small interfering RNA (siRNA) experiment

Synthetic shRNA targeting human *GADD45b* and non-targeting control siRNA (Invitrogen) were transfected into differentiated podocytes with Lipofectamine RNAi MAX reagent (Invitrogen, Carlsbad, CA). The target sequences of double-stranded nucleotides used for siRNA knockdown are 5′-ACGAGTCGGCCAAGTTGATGAATGT-3′ for GADD45B (1), 5′-CAGTCCTTCTGCTGTGACAACGACA-3′ for GADD45B (2), 5′-GAGGTGGCCAGCTACTGCGAAGAAA-3′ for GADD45B (3). A nonspecific, red fluorescence-labeled, double-strand RNA (BLOCK-iT Alexa Fluor Red Fluorescent Oligo (Invitrogen), was transfected in parallel as an siRNA-negative control. Synthetic shRNA transfection was performed according to the manufacturer's protocol. At 48 h after transfection, cells were treated with TGF-*β* and PAN for 24 h. The total protein extracts from the cells were used for western blot analysis.

### Western blot analysis of GADD45B protein expression

Cells were lysed in cold cell lysis buffer (50 mM Tris, 150 mM NaCl, 10 mM EDTA, 1% Triton X-100) containing protease and phosphatase inhibitors. The cell lysates (5 0μg) were heated for 5 min at 95 °C in sample buffer, separated on 10% SDS- polyacrylamide gel and transferred onto nitrocellulose membrane. After blocking with 5% non-fat dry milk, membranes were probed with rabbit polyclonal antibody to GADD45B and GAPDH (1 : 1000, Santa Cruz, Waltham, MA, USA). Membranes were incubated with HRP-conjugated anti-rabbit antibody (1 : 1000) and visualized by enhanced chemiluninecence detection. Each experiment was repeated for at least three times independently.

### Annexin V flow cytometric analysis of apoptosis

After the treatment, podocytes were collected, washed with ice-cold PBS twice, resuspended in 100 *μ*l of binding buffer, and then incubated with APC-conjugated Annexin V(1 : 20) and 7-AAD (1 : 10) (Liankebio, Hanzhou, China) at room temperature for 15 min, followed by analysis with FACScan using Cellquest software (Becton Dickinson, Franklin Lakes, NJ, USA). Each experiment was repeated for at least three times independently.

### ROS assay

Human podocytes were treated with 50 *μ*g/ml PAN and the intracellular production of ROS was assayed using the fluoroprobe CM-H2DCFDA (chloromethyl-2, -dichlorodihydrofluorescein diacetate as described previously.^7^ Each experiment was repeated for at least three times independently.

### Western blot analysis of MAPK activation

MAPK activation was analyzed with western blot as described before.^38^ Primary antibodies were diluted in TTBS and added as follows: anti JNK and anti-phospho-JNK, anti ERK 1/2, anti-phospho-ERK1/2, anti p38, antiphospho-p38 (Santa Cruz Biotechnology, Santa Cruz, CA, USA; mouse monoclonal IgG, dilution 1 : 200); anti GAPDH (Beyotime Biotechnology; mouse monoclonal, dilution 1 : 10 000). Each experiment was repeated for at least three times independently.

### Statistical analysis

Statistical analyses were performed with SPSS software (version 11.5). Results were expressed as means±S.D. Two groups were compared using the unpaired *t*-test. Comparison of three or more groups was performed by one-way ANOVA. *P*<0.05 was considered statistical significance, and *P*<0.01 was considered highly statistical significance.

## Abbreviations

DMSO: dimethyl sulfoxide
ERK: extracellular signal-regulated kinase
GADD45: growth arrest and DNA damage gene 45
Gal4: galactose-responsive transcription factor GAL4
JNK: c-Jun N-terminal kinase, JNK
lfabp: liver fatty-acid binding protein
LPS: lipopolysaccharide
MAPK: mitogen-activated protein kinase
MO: morpholino
MTZ: metronidazole
NTR: nitroreductase
PAN: puromycin aminonucleoside
ROS: reactive oxygen species
TGF-*β*: transforming growth factor-*β*
UAS: upstream active sequence
VDBP: vitamin D-binding protein
