# Supplementary material for: GADD45B mediates podocyte injury in zebrafish by activating the ROS-GADD45B-p38 pathway
Source: Cell Death Dis. 2016 Jan 21;7(1):e2068–. doi: 10.1038/cddis.2015.300 (PMC4816163; doi:10.1038/cddis.2015.300)
Supplement: Supplementary Information [file cddis2015300x1.doc]

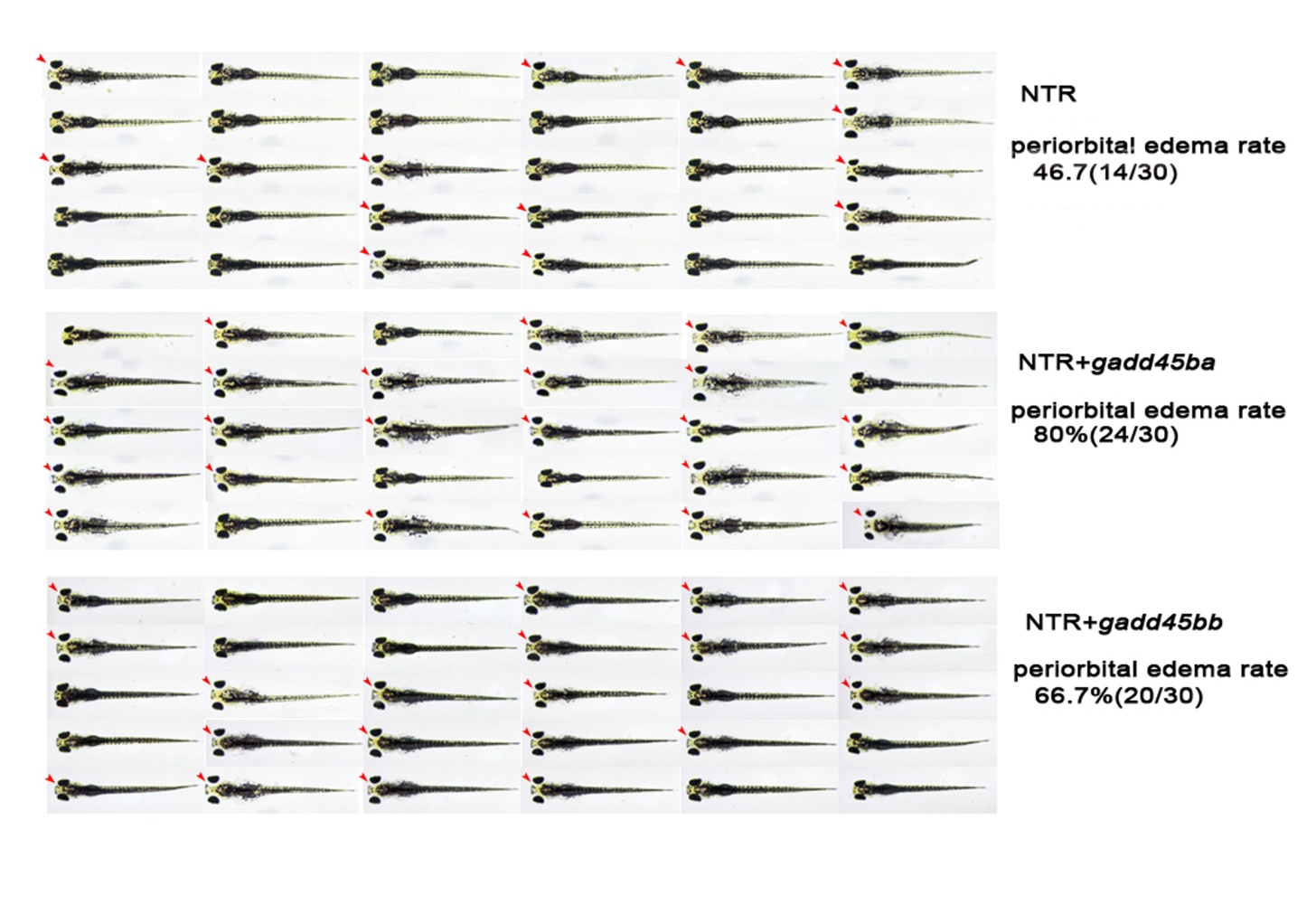


**Fig S1 Figures of each individual 5 day-old larva following 100 μM MTZ treatment for 48 hours showing the occurrence of periorbital edema ( Arrowhead mark larvae with periorbital edema).**

**Supplementary Table 1:** Quantitation of the periorbital edema phenotype after MTZ treatment.

|  | MTZ concentration | | | |
| --- | --- | --- | --- | --- |
|  | 60μM  (n=30) | 80μM  (n=30) | 100μM  (n=30) | 120μM  (n=30) |
| NTR | 9.0±3.6% | 26.3 ±5.1% | 56.0±8.7% | 74.3±9.2 |
| NTR and gadd45ba | 30.3±10.0%* | 61.7±9.9%** | 84.3±5.9%**^#^ | 94.7±4.6%*^#^ |
| NTR and gadd45bb | 23.3±11.5% | 45.0±7.0%* | 73.0±3.6%* | 86.2±7.7%* |

**P*<0.05, ***P*<0.01 vs NTR group, # *P*<0.05 vs NTR and gadd45bb group determined by t-test

**Supplementary Table 2: Quantitation of proteinuria (ng/ml) in zebrafish with podocyte specific overexpression *gadd45ba/gadd45bb* after MTZ treatment.**

|  | MTZ(100μM)  (n=10) | MTZ(120μM)  (n=10) |
| --- | --- | --- |
| NTR | 0.94±0.45 | 7.23±1.35 |
| NTR and gadd45ba | 5.69±0.99*^Δ^ | 20.26±1.65*^Δ^ |
| NTR and gadd45bb | 2.06±0.61 | 11.07±2.01* |

**P*<0.01 vs NTR group; ^Δ^ *P*<0.01 vs NTR and gadd45bb group.

The proteinuria levels are less than 1ng/ml in all the groups treated with 60μM and 80μM MTZ.

**Supplementary Table 3: Quantitation of the fish with *gadd45ba*/*gadd45bb*** knockdown by morpholino exhibiting periorbital edema after MTZ treatment.

|  | MTZ concentration | | | | |
| --- | --- | --- | --- | --- | --- |
|  | | 60μM  (n=30) | 80μM  (n=30) | 100μM  (n=30) | 120μM  (n=30) |
| NTR | | 9.3±4.0% | 23.7 ±5.1% | 58.3±8.0% | 74.7±5.5 |
| NTR+gadd45ba MO | | 6.3±3.5%* | 13.0±3.1%* | 33.3±6.5%* | 57.7±8.1%* |
| NTR+gadd45bb MO | | 5.4±1.5% | 15.3±4.7% | 41.6±8.5% | 64.3±2.3%* |
| NTR +control MO | | <0.1 | <0.1 | <0.1 | <0.1 |

**P*<0.01 vs NTR group

**Supplementary Table 4: Quantitation of proteinuria (ng/ml) in zebrafish with *gadd45ba/gadd45bb knockdown* by morpholino after MTZ treatment.**

|  | MTZ(100μM)  (n=10) | MTZ(120μM)  (n=10) |
| --- | --- | --- |
| NTR | 1.33±0.46 | 8.04±1.05 |
| NTR+gadd45ba MO | 0.77±0.14 | 2.46±0.14** |
| NTR+gadd45bb MO | 0.83±0.40 | 4.97±0.95* |
| NTR +control MO | 1.71±0.19 | 9.27±1.04 |

**P*<0.05, ** *P*<0.01 vs NTR group

**Supplementary Table 5** List of primer sequences

| Gene | Application | Forward primers | Reverse Primers |
| --- | --- | --- | --- |
| *gadd45ba* | ORF | ttGGTACCatgaccctggaagaagtcgttg | aGAATTCtcagcgttcttgcagggacaG |
| *gadd45bb* | ORF | ttGGTACCatgactctggaggaagttgttg | aGAATTCtcagcgctcttgcagg |
| *gadd45ba* | qRT-PCR | ACTGCATCCTCGTCACTAACTC | TTTTGCAACGGCTCTCCTCA |
| *gadd45bb* | qRT-PCR | TGTTACTAACCCCCAAGCCG | GGCAATAGAAGGCACCCAC |
| *podocin* | qRT-PCR | CTCTAGCAGCACGGTTGTAAA | AGGTCCCTCAGTCTCCAATAA |
| *podocin* | RT-PCR | CAGTGTGAGGGAACGGATAAA | ATGCTAGCGAAGGAGGAATAAC |
| *flia1* | qRT-PCR | GCCATTAAGGAGTACGGTCTTC | GAGTCTCAGGAAGTCGTCTTTG |
| *cdh17* | qRT-PCR | CAAGATGGAGGCTGAGATGAAG | CATGTCCTGAAGGCAGATGAA |
| *ef1a* | qRT-PCR | CTGGAGGCCAGCTCAAACAT | ATCAAGAAGAGTAGTACCGCTAGCATTAC |
